# Shell Vial culture Assay for the rapid diagnosis of Japanese encephalitis, West Nile and Dengue-2 viral encephalitis

**DOI:** 10.1186/1743-422X-3-2

**Published:** 2006-01-06

**Authors:** Rangaiah S Jayakeerthi, Raghava V Potula, S Srinivasan, S Badrinath

**Affiliations:** 1 Department of Microbiology, Jawaharlal Institute of Post-graduate Medical Education and Research, Pondicherry – 605 006, India; 2 Department of Pediatrics, Jawaharlal Institute of Post-graduate Medical Education and Research, Pondicherry – 605 006, India

## Abstract

**Background:**

Encephalitis caused by flaviviruses, Japanese encephalitis virus (JEV) and West Nile virus (WNV) is responsible for significant morbidity and mortality in many endemic countries. Dengue-2 (Den-2) virus is a recent addition to the list of encephalitogenic viruses, after its Central Nervous System (CNS) invasion capability has been established. There is a wide array of laboratory tools that have helped us not only in the diagnosis of these conditions but also in understanding their pathogenesis and pathology. However, there are no reports of Shell Vial Culture (SVC), a centrifuge enhanced tissue culture assay that has revolutionized viral culturing in terms of rapidity and sensitivity being optimized for these flaviviral encephalitic conditions. The present study is an attempt to standardize and evaluate the usefulness of SVC for the laboratory diagnosis of JE, WN and Den-2 encephalitis cases and to compare it with Indirect Immunofluorescence (IIF) technique that detects cell associated virus antigen. Analysis of the various clinical parameters with respect to viral etiology has also been carried out.

**Results:**

Pediatric patients constituted the major group involved in the study (92%). Etiological diagnosis of viral encephalitis could be established in twenty nine (58%) patients. JE encephalitis was the commonest with 19 (39%) cases being positive followed by, WN (9 cases-18%) and Den-2 (one case). IIF test could detect antigens of JE, WN and Den-2 viruses in 16(32%), 7(14%) and 1 case respectively. Shell vial culture assay picked up all cases that were positive by IIF test. In addition, SVC assay could detect 3 and 2 more cases of JE and WN encephalitis respectively, that were negative by the IIF test.

**Conclusion:**

Shell vial culture is a rapid and efficient tool for the etiological diagnosis of JE, WN and Den-2 encephalitis cases. Early, prompt collection, transport and processing of the CSF samples, would make SVC a better method for the rapid diagnosis of these flaviviral infections.

## Background

The family flaviviridae encompasses viral agents, which are an important cause of arthropod-borne encephalitis in humans. Japanese Encephalitis (JE) virus is the most common among them in the world [1]. In India, JE, the leading cause of viral encephalitis, has been endemic in South India, since 1978. The mortality of this infection varies from 20–40% in different parts of India [2].

West Nile virus, a close relative of JEV, usually causes a mild febrile illness in humans, that may sometime end in encephalitis [3]. High morbidity and mortality experienced during the epidemics of West Nile encephalitis in USA [4] and Israel [5] is an example for the severity of disease.

Dengue-2 (Den-2) virus is another flavivirus that is primarily associated with a febrile illness (Dengue fever), Dengue hemorrhagic fever syndrome (DHS) and Dengue shock syndrome (DSS). It is a recent addition to the list of encephalitogenic viruses, after its neurovirulence has been proved by isolation of the virus from CSF, demonstration of specific IgM antibodies and viral genome detection in CSF by RT-PCR [6].

Infections by JE, WN and Den-2 viruses are prevalent in India. Earlier studies from our institution have shown high prevalence of JE infection and also serological evidence of WN and Dengue-2 infections in Pondicherry and surrounding areas [7].

Various techniques have been tried, standardized, evaluated for the laboratory diagnosis of infections due to JE, WN and Den-2 viruses. The rapid laboratory tools that can be of help in clinical situations of viral encephalitis include, demonstration of viral antigen, detection of specific IgM antibody and genome detection [8]. Though RT-PCR in CSF is useful for diagnosis of WNE, detection of viral genome is of limited use, and the diagnostics have to be based, or at least supplemented, by serology for Japanese encephalitis and Dengue viruses [9]. Isolation in cell culture, though cumbersome, time consuming and less sensitive is still considered the gold standard for the diagnosis of flaviviral encephalitis [8].

Shell vial culture is a modification of the conventional cell culture technique for rapid detection of viruses in vitro [10]. The technique involves inoculation of the clinical specimen on to cell monolayer grown on a cover slip in a shell vial culture tube, followed by low speed centrifugation and incubation. This system works on the principle that the low speed centrifugation enhances viral infectivity to the susceptible cells. It is thought that the minor trauma to the cell surface produced as a result of low speed centrifugation mechanical force enhances the viral entry in to the cells, which in turn reduces the total time taken for the virus to produce infection of cells [11]. Originally the SVC was described for murine cytomegalovirus [12]. Later, this principle was employed in the field of medical microbiology for the isolation of *Chlamydia trachomatis *from the human genital tract [13]. Rapid identification of cytomegalovirus in infected human urine specimens using Human Diploid Fibroblast (MRC-5) cells demonstrated its usefulness in diagnostic virology [14]. The rapidity of the technique without any compromise on sensitivity has made SVC very popular in the field of clinical virology. The same technique has been employed for the identification of other medically important viruses such as Herpes Simplex Virus (HSV), Varicella-Zoster Virus (VZV), Adenovirus, Influenza A&B, Para influenza 1,2,3 and Respiratory Syncitial Virus (RSV) by various groups of workers successfully [15].

## Results

### Age

46 (92%) patients were from pediatric age group ranging from 18 months to 12 years with a mean age of 7 years.

### Sex

Predominant sex was male with a M: F ratio of 1.7:1.

### Clinical presentation

Fever, Seizures and Altered Sensorium were the clinical symptoms with which most of the patients sought medical help. Fever (98%) was the commonest initial manifestation followed by altered sensorium (92%) and seizures (66%). Headache and Vomiting were present in 4 (8%) and 7(14%) cases respectively.

### Duration of hospital stay

30 (60%) patients stayed only for a week or less period of time in the hospital. Only 8 (16%) patients stayed for 3 weeks or more. Mean duration of hospital stay was 7.5 days.

### Virological studies (Table 1.)

29 (58%) specimens in the study were positive for one of the three flaviviruses studied either by IIF or shell vial culture or both. JEV was the commonest (19 cases-38%), followed by WNV (9cases-18%). Den-2 was detected only in one case.

**Table 1:** Comparison of indirect immunofluorescence (IIF) and shell vial culture (SVC) techniques

SVC	IIF				
		
	Negative No. (%)	Positive for JE antigen No. (%)	Positive for WN antigen No. (%)	Positive for Den-2 antigen No. (%)	Total No. (%)
Negative	21 (100)	-	-	-	21 (42)
Positive for JE virus	3 (15.8)	16 (84.2)	-	-	19 (38)
Positive for WN virus	2 (22.2)	-	7 (77.8)	-	9 (18)
Positive for Den-2 virus	-	-	-	1 (100)	1 (2)

Total	26 (52)	16 (32)	7 (14)	1 (2)	50 (100)

### Indirect immunofluorescence technique

IIF technique could detect the three flaviviruses studied in 24 (48%) cases. Out of this, 16 (32%) were JE, followed by WN and Den-2 in 7 (14%) and 1 (2%) specimens respectively.

### Shell vial culture technique

SVC could pick up all the cases positive by IIF technique. Additional 3 and 2 cases of JE and WNE could be diagnosed by SVC that were negative by IIF test. So, SVC could establish viral etiology in 29 (58%) of the cases studied. JEV was positive in 19 (18%) of cases followed by 9 (18%) WNV and Den-2 virus (one case)

### Distribution of JE, WN and Den-2 viruses among the different age and sex groups

Among the four adults (age > 12 years) included, only one case was positive for JE. Rest three were negative for all the flaviviruses studied. Twenty eight (60.8%) patients from pediatric age group (n = 46) were positive for one of the 3 viruses studied. JE was found in 18 (64.3%) cases followed by WN in 9 (32.2%) cases and Den-2 in one (3.5%) case. Seventeen (53.1%) out of 32 male patients studied were positive for one of the three viruses studied. JE was found in 11 (64.7%) cases followed by WN in 5 (29.3%) cases and Den-2 in one (6%) case. Out of the 18 (36%) total female patients in this study, 8 (44.4%) patients were positive for JE and 4 (22.2%) patients for WN. No Den-2 was detected in this group.

### Focal neurological deficits

Three different types of focal neurological deficits (FND) were identified in the patients studied. Commonest was facial palsy, which was seen in 6 (12%) cases, all of them were positive for JEV infection. Hemiparesis was found in 3 (6%) cases that were positive for JEV infection. Hemiplegia was seen in one (2%) patient with JEV infection. Hence, all the FND were associated with JEV infection. No FND were associated with WN and Den-2 viral infections. A total of 9 (18%) patients had an associated FND. One patient was having hemiplegia associated with facial palsy. No FND were found among the patients negative for the 3 viruses studied.

### Clinical outcome (Table 2.)

Eight patients positive for JEV expired with a case fatality rate (CFR) of 50% and the rest 8 (50%) had a complete clinical recovery. Five patients who were positive for WNV expired (CFR = 62.5%) and the rest 3 recovered. One case, which was positive for Den-2 virus, showed complete recovery. Three (14.3%) among the 21 patients who were negative for all 3 viruses expired, whereas 18 (85.7%) of them showed clinical improvement. Four patients were discharged against medical advice; hence the outcome of these cases could not be analyzed.

**Table 2:** Clinical outcome among the cases positive for JE, WN and DEN-2 viruses

Viruses	Expired No. (%)	Recovered No. (%)	Total No. (%)
JE	8 (50)	8 (50)	16 (34.8)
WN	5 (62.5)	3 (37.5)	8 (17.3)
Den-2	-	1 (100)	1 (2.2)
Negative	3 (14.3)	18 (85.7)	21 (45.7)

Total	16 (34.8)	30 (65.2)	46* (100)

*Four out of fifty patients included in the study left the hospital against medical advice, the clinical outcome of whom is not known. Hence, these 4 cases were excluded from the analysis of clinical outcome.

## Discussion

Japanese encephalitis virus is the most important cause of epidemic encephalitis worldwide with an estimated 30,000–50,000 cases annually [16]. The geographical area affected by the virus is expanding, and despite the availability of vaccines, JE is a growing public health problem [17,18]. West Nile virus, until recently being a relatively benign virus, causing epidemics of a fever-arthralgia-rash syndrome, and only occasional CNS disease [19] has disproved this belief with the recent outbreaks of WN encephalitis in Romania and New York [20,21]. These viral encephalitic conditions are acute in onset and may mimic other acute infectious conditions of CNS such as, Tubercular meningitis, cerebral malaria and other viral encephalitis [22]. The need for an accurate and rapid diagnostic test is much sought for.

Conceptually, the most rapid diagnosis of an arbovirus infection can be made by direct detection of virus antigen or nucleic acid in clinical specimens. Rapid serologic diagnostic tests, such as Enzyme Immuno-Assay (EIA) can provide strong presumptive etiologic evidence, if specific IgM is detected in the acute-phase serum or CSF specimen. Detection of IgM is not always evidence of current infection with certain arboviruses, especially flaviviruses, which can induce persistent IgM production [23,8]. Genome detection, though very useful in diagnosing WN encephalitis, is of limited value in diagnosing JE and Dengue encephalitis [9].

Virus isolation, though more specific compared to antigen detection, is less sensitive and time consuming requiring minimum of 3–7 days compared to 5–6 hours required for antigen detection by indirect immuno- fluorescent technique [24]. Shell vial culture, a modification of conventional cell culture technique works on the principle that centrifugation mechanical force enhances the viral infectivity to the susceptible cells [11]. This technique has been used for the rapid diagnosis of infections by various viruses such as cytomegalovirus, Herpes Zoster, Mumps, Measles and respiratory syncytial virus [15]. In all these studies, shell vial culture technique has been shown to increase the rate of isolation of the viruses without any compromise on the specificity. Shell vial culture technique also has been shown to significantly reduce the time taken as compared to conventional cell culture technique.

In the present study that included 50 patients, males were 32 (64%), and females were 18(36%) with a male to female ratio of 1.7:1. Pediatric age group (0–12 years) was the predominant one compared to the adults. This predominant involvement of pediatric age group (92%) and the Male:Female ratio of 1.7:1 are in accordance with the age and sex distribution of the earlier epidemics of viral encephalitis in the endemic areas including Pondicherry and Tamilnadu [25]. Viral etiology could be proved in 29(58%) out of 50 cases, of which 28 (96.5%) belonged to the pediatric age group. JEV was the commonest etiological agent (38%) followed by WNV (18%) and Den-2 virus in one case. These findings suggest a high incidence of JE and WN encephalitis in this region of South India. Endemicity of JEV in South India is a known fact. There have been earlier documented cases of WN encephalitis from South India [26,27]. A recent report from India [28] documents 88 sporadic cases of WN infection including 7 cases of WN encephalitis. This suggests that WNV is active and prevalent in India. Though Dengue infection is present since ancient times in India, documented encephalitic form is rare. Isolated reports of Dengue encephalitis from India [29] suggest that it might be an under -diagnosed clinical entity, because of lack of proper laboratory facilities.

A total of 9 (65.5%) out of 19 patients positive for Japanese encephalitis virus had neurological deficits. Focal neurological deficits occurred in 6(31.5%) patients who recovered from Japanese encephalitis. The neurological deficits that were noted in the study group were facial palsy (n = 6), hemiparesis (n = 3) and hemiplegia (n = 1). All the neurological deficits were found in patients positive for Japanese encephalitis virus. In one case facial palsy was associated with hemiplegia. No neurological deficits were noticed in patients positive for West Nile or Den-2 virus infections. It is interesting to note the absence of neurological deficits like flaccid paralysis in WN encephalitis cases that is described in outbreaks of WN virus infection in the United States[30,31].

Among the 50 patients included in the study, 4 (8%) patients left against medical advice, so the clinical outcome of these cases is not known. Out of the remaining 46 (92%), 16(34.8%) expired and the rest 30(65.2%) had a clinical recovery with or without neurological sequelae. 8 patients positive for Japanese encephalitis virus expired with a case fatality rate (CFR) of 50%. In the present study, West Nile encephalitis had a CFR of 62.5%. Dengue-2 infection was detected only in one patient, who showed complete clinical recovery. This high case fatality rate among children with West Nile encephalitis is in contrast to other published reports where in advanced age is the most important risk factor for morbidity and mortality. It is unclear if this high mortality rate among the children is due to a highly virulent strain of the virus.

Indirect immunofluorescence test, the best-studied antigen detection method for the diagnosis of viral encephalitis by flaviviruses especially Japanese encephalitis [32], could detect the viral antigens in 24(48%) cases. This finding correlates with the earlier IIF studies for the diagnosis of viral encephalitis [32,33].

Shell Vial Culture could demonstrate viral etiology in all the 24 cases positive by IIF. In addition shell vial culture could detect the virus in 5(10%) more cases, which IIF failed to detect. Therefore, the technique could establish viral etiology in 29(52%) cases compared to 24(48%) by IIF. The significant reduction in the time taken (36 hours), compared to conventional cell culture technique (3–7 days) is an important advantage, which could be due to the hastening of viral entry into the cells of monolayer, as hypothesized. In order to ensure a rapid and precise diagnosis, early, prompt collection, transport and processing of the CSF samples becomes mandatory.

## Conclusion

Shell vial culture is a rapid and efficient tool for the etiological diagnosis of JE, WN and Den-2 encephalitis cases. It is more sensitive than the Indirect Immunofluorescence technique, which is a widely used rapid diagnostic method for the diagnosis of viral encephalitis. Early, prompt collection, transport and processing of the CSF samples, would make SVC a better method for the rapid diagnosis of these flaviviral infections.

## Materials and methods

### Specimens

Cerebrospinal fluid obtained by lumbar puncture from 50 patients admitted in pediatric and medical wards, JIPMER hospital, Pondicherry with a provisional clinical diagnosis of viral encephalitis constituted the study material. The group included 46 pediatric patients aged between 18 months and 12 years and 4 adults with age ranging from 13 to 40 years. All the patients were from Pondicherry and the neighboring districts of Tamil Nadu State.

### Cell line

Porcine Kidney (PS) cells (supply No 3109 A) obtained from National Center for Cell Sciences, Pune, were used in the study. Cells were grown in plastic tissue culture flask (NUNC, Denmark) and Roux bottles at 37°C.

### Medium

Eagle's modified MEM (AT 017, autoclavable) with Earle's salts, NEAA, phenol red without L-glutamine, NaHCO_3 _and antibiotics (Hi-media Lab. Pvt. Limited, Mumbai) was used. L glutamine (3%), vitamin concentrate, glucose (10%), fetal calf serum (10%) and the mixture of antibiotics (Penicillin 100 U/ml, Gentamicin 4 mg/ml, Streptomycin 100 μg/ml, Ciprofloxacin 2 mg/ml and Amphotericin-B 5 mg/ml) were added after the reconstituted Eagle's modified MEM was autoclaved.

### Shell vials

Pre-sterilized flat-bottomed cylindrical shell vials (4.5 cm × 1.5 cm), with a cover slip of 13 mm diameter obtained from Flow Laboratories, Scotland.

### Mouse Ascitic Fluid (MAF)

The lyophilized MAF against JEV (IPF, M-61106), WNV (825605-2) and Dengue virus (M-90450) obtained from National Institute of Virology, Pune were reconstituted with phosphate buffered saline (pH 7.2) and used for staining CSF smears and cell monolayers.

### Rabbit IgG FITC conjugate

Rabbit IgG FITC conjugate (Sigma product No. F7256) was used for secondary staining of the CSF smears and cell line monolayer of the shell vial.

### Controls

JEV (NIV strain P 20778), WNV (NIV strain G 22886), Dengue-2 (NIV strain P 23085) infected mouse brains obtained from National Institute of Mental Health and Neurosciences, Bangalore were blind passaged in suckling mice by intracerebral inoculation.

Subsequently the strains were adapted to porcine kidney cell line in the laboratory by serial blind passage. Porcine kidney cell line infected with JE, WN and Dengue-2 viruses stained by indirect immunofluorescence served as positive controls for the IIF study with clinical specimens.

### Procedure for IIF

Glass slides (75 × 25 × 1.35 mm, World Star micro slides) were washed with soap water, kept immersed in Teepol solution overnight, washed and autoclaved. Prior to the making of smears, these slides were treated with 1:1 mixture of Methanol-Ethanol for 30 mins in a Coplin jar, air-dried and used.

Using a cytospin system (Auto smear, CF-12DE, SAKURA, Japan) three smears were made on separate glass slides from each of the 50 specimens, one each for JE, WN and Den-2 virus respectively. 150 μl of CSF specimen was used to prepare one cytospin smear. The cells were sedimented using the cytospin at a speed of 1000 rpm for a time period of 10 mins.

Smears were air dried, fixed with chilled acetone for 30 mins. 20 μl of 1:10 MAF against JE, WN and Den-2 were added to smears 1,2&3 respectively and incubated in a humid chamber at 37°C for 30 mins, followed by several washes with PBS (pH 7.2). 20 μl of 1: 10 Rabbit anti-mouse IgG FITC conjugate was added to each of these smears and incubated at 37°C in a humid box for 30 mins. Smears were washed several times over a period of 10 mins using PBS (pH 7.2). Smears were then dried and mounted with buffered glycerol (Bartels buffered glycerol mounting medium pH 8–8.4, B1029-45 B, Baxter diagnostic Inc.) and observed under fluorescence microscope (Olympus, Japan) for intracytoplasmic apple green fluorescence.

Positive and negative controls were included in every run of the assay for comparison. Smears were examined by two independent examiners and recorded.

### Standardization of Shell vial culture

After adaptation of JE, WN and Dengue-2 viruses (NIV strains) to the porcine kidney cell line, the strains were used for the standardization of shell vial culture. Porcine kidney cell monolayers were grown on the cover slips of shell vials. The NIV strains were used to infect the monolayers. After centrifugation at 1000 rpm for 45 mins, the shell vials were incubated at different time periods of 12, 24, 36 and 48 hrs at room temperature. Cell monolayers were fixed with chilled acetone and stained by indirect immunofluorescence technique as described below. It was found that early best results were obtained after 36 hours of incubation and this served as positive controls for the shell vial assay in this study.

### Procedure for Shell vial assay

1 ml of the porcine kidney cells suspension (4 × 10^5 ^cells/ml) in growth medium was added to each shell vial and incubated at 37°C till the confluent monolayer was formed. Shell vials were numbered 1,2 & 3 and the virus growth medium was removed from them. 300 μl of the specimen was inoculated into each of these shell vials 1,2 & 3 that were later stained by IIF technique for JE, WN and Den-2 viruses respectively. Shell vials were centrifuged at a speed of 1000 rpm for 45 mins at room temperature followed which 1 ml of maintenance medium was added in to each of the shell vials to incubate at 37°C for 36 hours. Maintenance medium was removed carefully from the shell vials and discarded. Monolayer was rinsed with PBS (pH 7.2) 3–4 times dried and fixed with chilled acetone for 30 mins. Coverslips from the shell vials 1,2 & 3 were stained by IIF method for JE, WN and Den-2 viruses respectively as described above. Smears were examined under fluorescence microscope by two independent observers for intra-cytoplasmic apple green fluorescence.

## List of abbreviations

JE: Japanese encephalitis

WN: West Nile

Den-2: Dengue-2

IIF: Indirect Immunofluorescence

SVC: Shell vial culture

## Competing interests

The author(s) declare that they have no competing interests

## Authors' contributions

JSR and RVP designed the study, did the IIF and SVC tests and documented the resultsSS collected CSF specimens, did clinical examination of the patients analysis of clinical outcome in the studied population

JSR and BS analyzed the test results and prepared the manuscript
